# Childhood adversity, mental health and suicide (CHASE): a methods protocol for a longitudinal case-control linked data study

**DOI:** 10.23889/ijpds.v5i1.1338

**Published:** 2019-04-02

**Authors:** N Dougall, J Savinc, M Maxwell, T Karatzias, RC O’Connor, B Williams, G Grandison, A John, H Cheyne, C Fyvie, JI Bisson, C Hibberd, S Abbott-Smith, L Nolan

**Affiliations:** 1 School of Health & Social Care, Edinburgh Napier University, Edinburgh, EH11 4BN, UK; 2 Nursing, Midwifery and Allied Health Professions Research Unit, Scion House, University of Stirling, Stirling, FK9 4LA, UK; 3 Institute of Health & Wellbeing, University of Glasgow, Glasgow, G12 8RZ, UK; 4 Swansea University Medical School, Swansea University, Swansea, SA2 8PP, UK; 5 The Rivers Centre, NHS Lothian, Edinburgh, EH11 1BG, UK; 6 Cardiff University School of Medicine, Cardiff University, Cardiff, CF24 4HQ, UK; 7 Faculty of Health Sciences & Sport, University of Stirling, Stirling, FK9 4LA, UK; 8 Child and Adolescent Mental Health Service (CAMHS), NHS Lothian, Edinburgh, EH10 5HF, UK; 9 Aberlour, Scotland’s children’s charity (SC007991), Stirling, FK8 2JR, UK

## Abstract

**Introduction:**

Suicide is a tragic outcome with devastating consequences. In 2018, Scotland experienced a 15% increase in suicide from 680 to 784 deaths. This was marked among young people, with an increase of 53% in those aged 15-24, the highest since 2007. Early intervention in those most at risk is key, but identification of individuals at risk is complex, and efforts remain largely targeted towards universal suicide prevention strategies with little evidence of effectiveness.

Recent evidence suggests childhood adversity is a predictor of subsequent poor social and health outcomes, including suicide. This protocol reports on methodology for harmonising lifespan hospital contacts for childhood adversity, mental health, and suicidal behaviour. This will inform where to 1) focus interventions, 2) prioritise trauma-informed approaches, and 3) adapt support avenues earlier in life for those most at risk.

**Methods:**

This study will follow a case-control design. Scottish hospital data (physical health SMR01; mental health SMR04; maternity/birth record SMR02; mother’s linked data SMR01, SMR04, death records) from 1981 to as recent as available will be extracted for people who died by suicide aged 10-34, and linked on Community Health Index unique identifier. A randomly selected control population matched on age and geography at death will be extracted in a 1:10 ratio. International Classification of Disease (ICD) codes will be harmonised between ICD9-CM, ICD9, ICD10-CM and ICD10 for childhood adversity, mental health, and suicidal behaviour.

**Results:**

ICD codes for childhood adversity from four key studies are reported in two categories, 1) Maltreatment or violence-related codes, and 2) Codes suggestive of maltreatment. ‘Clinical Classifications Software’ ICD codes to operationalise mental health codes are also reported. Harmonised lifespan ICD categories were achieved semi-automatically, but required labour-intensive supplementary manual coding. Cross-mapped codes are reported.

**Conclusion:**

There is a dearth of evidence about touchpoints prior to suicide. This study reports methods and harmonised ICD codes along the lifespan to understand hospital contact patterns for childhood adversity, which come to the attention of hospital practitioners.

**Key words:**

Childhood Adversity, Adverse Childhood Experiences, Mental Health, Self-harm, Suicide, Suicidality, Violence, Hospital episodes, Routine Data, Data Linkage, Study Protocol

## Introduction

In the UK, suicide is the leading cause of death for young people with devastating consequences for those affected. Suicide rates in Scotland are particularly tragic, being disproportionately higher than that of England, and especially so for young men [1]. In 2018 Scotland experienced a 15% increase in suicide from 680 to 784 deaths, with this increase especially marked among young people [2]. Alarmingly, the numbers of those dying in 2018 aged 15-24 increased by 53%, the highest seen since 2007 [2], while those aged 10-34 increased from 24.4% (161/659) to 30.8% (232/753) between 2014 and 2018 [2]. This five year increase reversed a long-term downward trend from a comparable peak of 39.8% (363/912) in 1993 [2].

This pattern was consistent across UK, where deaths by suicide increased by 11% in 2018 to 6,507, and for those aged 15-24 increased by 24%, reaching 730 deaths [3]. The UK and Scottish rate in 2018 for all ages was 11.2 and 14.6 per 100,000 inhabitants respectively, rising to 19.1 per 100,000 inhabitants for those aged 15-24 in Scotland [3]. These recent data suggest a trend for increased vulnerability in younger people in the UK, and are associated with corresponding increases of self-harm since 2010, a strong risk factor for suicide [4]. Deliberate acts of self-poisoning and self-injury are increasingly becoming more common among young people, with UK psychiatric morbidity data for 2014 reporting that 26% and 10% of women and men aged 16-24 have reported they have self-harmed [5].

One potential way to address this problem is to intervene earlier in the lives of people most at risk, and prior to suicidal behaviour. In order to do this, an exposure requires to be identified that not only acts as a risk factor for suicide, but also to mental health problems which emerge earlier in the lifespan, i.e. in childhood or young adulthood. It is now accepted that those who are exposed to adversity in childhood are at much increased risk of poor social and health outcomes, and in particular mental health and suicidal behaviour [6–8].

Childhood adversity can be the consequence of harm experienced at an individual level, including those first reported in the Adverse Childhood Experiences (ACEs) study [9]. The ACEs study defined adverse events in childhood as a restricted set of traumatic experiences, such as sexual, emotional and physical abuse, neglect and maltreatment [9], an approach considered to be overly reductionist as it does not consider physical or emotional harm perpetrated via other family, social and geographical factors [10,11]. In this debate social inequality plays an important role, 24% of Scotland’s children are estimated to be living in relative poverty [12], and one Scottish study estimated the majority of children experienced one ACE by age 8, concluding that ACEs are highly correlated with socioeconomic disadvantage in the first year of life [10].

A steep social gradient is also associated with suicide, with more people who take their own lives being from deprived communities [13]. A recent systematic review of 28 studies concluded that experience of adversities was significantly related to youth suicidal behaviour, and confirmed a strong dose-relationship between numbers and types of adversity, and increasing suicidal behaviour [14].

Although it is known that childhood adversity is a risk factor for suicide, current evidence is insufficiently detailed in two ways to develop targeted, effective and implementable approaches to prevention. Firstly, given that adverse life events include a wide range of experiences there is a need to identify what types are most likely to lead to mental health problems and suicidal behaviour. Secondly, given that a dose response relationship exists, there is also a need to know what number and/or combination of experiences is most likely to lead to mental health problems and suicidal behaviours. Uncovering patterns of healthcare contact may afford clearer scenarios to intervene earlier in the lives of those most at risk. This recent review could not address these points as a meta-analysis was not possible due to high levels of heterogeneity, and most included studies were cross-sectional, or retrospective and relied on recall which is prone to bias [14].

Given this important gap in knowledge, robust longitudinal studies are needed to explore causal mechanisms. There has been no prospective longitudinal lifespan study on adverse life events in childhood and later suicidal behaviour. Such a study would provide robust results, but take decades to complete. Linked population data studies offer powerful ways of advancing our understanding of individuals and societies [15]. In Scotland it is possible to summarise hospital records, which do not suffer from recall bias, and follow individuals along their life-span from 1981 [16]. Hospital records are coded by International Classification of Disease (ICD) codes; a number of ICD codes are available that are indicative of childhood adversity. Although presentations for e.g. violence and maltreatment are likely under-recorded, it is plausible that those serious enough to require hospital attention are associated with severity, necessitating attendance at Emergency Departments and/or requiring in-patient care.

For the purposes of this study, the working definition of childhood adversity will be as wide a typology as possible, encompassing individual-level codes which are on a continuum of definite, probable and suggestive of violence and maltreatment, and using area level variables of deprivation and urban-rural indicator derived from individuals’ postcodes. Key studies are available which have operationalised working definitions of childhood adversity, along with published ICD code lists [17–20].

Therefore, the aims of this study are two-fold: Firstly, to investigate patterns of lifespan contacts for childhood adversities stratified by deprivation as recorded in hospital records, and ascertain their relationship with mental health and suicidal behaviour, prior to suicide in young adults. Secondly, to explore via multi-agency stakeholder workshops, and in conjunction with experts by lived experience, how best to use insights revealed in the data in developing more focussed upstream interventions. The two aims of this study are underpinned by five research questions (table 1).

**Table 1: Study research questions:** 

Research questions
1) What is the relationship between number and type of childhood adversities and suicide, stratified by age and gender, and if possible by deprivation code? (Aim 1).
2) What is the relationship between type and number of childhood adversities, subsequent mental health, self-harm, self-poisoning admissions prior to suicide? (Aim 1).
3) Can a dose-response relationship of number of childhood adversities with suicidal behaviour be confirmed, and can type of childhood adversity be ranked as having impact on later life? (Aim 1).
4) Are maternal records which are linked to the child records sufficiently detailed and data-rich to be used as an indicator of maternal adversity, and if so, how does maternal adversity affect offspring mental health and suicidality? (Aim 1)?
5) Are the results of this study rich enough to be used to inform the development of an intervention? (Aim 2).

## Methods

### Study design and setting

This study will follow a case-control design using routine data. This design will make efficient use of existing data to compare people with the outcome of suicide (‘cases’) versus people who are ‘controls’. Retrospective comparisons will be made on the frequency in hospital records of childhood adversity present in each group; this will be used to explain any relationship if it exists between adversity in childhood, mental health, suicide attempts (self-harm) and suicide.

This study will be overseen by a Study Steering Group (SSG) with a range of backgrounds, comprised of academics, clinicians and charitable sector experts. These include population data science, statistics, suicidology, health intervention development, medicine, midwifery, clinical psychology, psychiatry, and child and adolescent mental health nursing. The study will be conducted in partnership with ‘Aberlour’, Scotland’s children’s charity that aims to support children, young people and their families with early intervention (https://www.aberlour.org.uk/). Additional lived experience perspectives will be provided via the Mental Health Foundation, (https://www.mentalhealth.org.uk/scotland) and the Violence Reduction Unit (http://actiononviolence.org/).

There are two stages to this study: stage 1 (aim 1; RQs 1-4), consisting of obtaining permissions to access data, cleaning and analysis of data remotely via the ‘Safehaven’ to ensure confidentiality of records, and stage 2 (aim 2; RQ5), consisting of the workshops and any intervention development work. 

### Datasets

Datasets to be used will be drawn from the following:

1. General Hospital Scottish Morbidity Records (‘SMR01’).
2. Psychiatric Hospital Records (‘SMR04’).
3. Birth record and maternity information (‘SMR02’).
4. Mother’s linked hospital records (deaths/ ‘SMR01’/ ‘SMR04’) to ‘SMR02’
5. National Records of Scotland death certificates (NRS).

Scotland is a country with a reasonably stable population of about five million. NHS healthcare is a national single state provider administered by the Scottish Government with very little private healthcare provided (2%), and is therefore representative of the population as a whole. It is free to access at the point of use, funded by general taxation, and the national Scottish Morbidity Record (SMR) datasets have been routinely collected and warehoused by the NHS Information Services Division since 1981 [16]. Every person born or registered with a General Practitioner in Scotland is allocated a unique ten digit ‘Community Health Index’ (CHI) number and entered on a national register, representing between 96.5-99.9% of the Scottish population [16]. Each SMR data entry (datasets 1 to 4) is recorded by a CHI number along with detailed individual-level information on variables such as: main and secondary diagnoses on admission and discharge, dates of admission and discharge, length of continuous episode of care (‘spell’), discharge destination, admission and discharge type, etc. Missing data proportions vary according to the dataset, variables, and year of capture and form a substantive part of data preparation before analysis. The CHI number thus enables healthcare records to be linked through time, between locations, and between datasets using direct matching and probabilistic matching, including NRS vital events death registrations (dataset 5), [16].

### Data linkage

Scotland does not have a single data warehouse. NHS and NRS data controllers assess research proposals and decide whether they are in the public interest and meet legislative requirements. Once permission is granted, data under the responsibility of the data controllers are brought together and linked on a project-by-project basis, facilitated by direct matching using the CHI unique identifier [16]. This study will be supported by the electronic Data Research Innovation Service (eDRIS), NHS ISD, Scotland. eDRIS have confirmed data availability, will provide the de-identified linked data and facilitate safe access via the secure National Safehaven environment, 9 Bioquarter, Edinburgh.

### Study Population

For the purposes of the case-control study, ‘cases’ are defined as data related to people who died by suicide or by undetermined intent at age ≤10 & ≥34 between 1991 and as recent as data are available, thus permitting lifespan records to have accrued for the first observed deaths at age 10 in 1991. The ‘controls’ are randomly selected from the population and matched on age, gender, geographically matched on postcode, being alive at the time of death of the ‘cases’, and following the rationale of a previous national suicide information database [21].

Using Scottish vital events data, deaths by suicide or of undetermined intent aged ≤10 & ≥34 between 1991 and 2015 numbered 6,907. Previous research by the study applicants on linked hospital records for people who died by suicide from 1980-2009 demonstrated >85% of death records were associated with a valid NHS number, and >75% had linkable hospital data; some 12,000 people were found to have 85,000 episodes of care [22]. Therefore, we expect many thousands of episodes in this study.

**Figure 1: Flow diagram of mothers and babies eligible for inclusion in study population:**
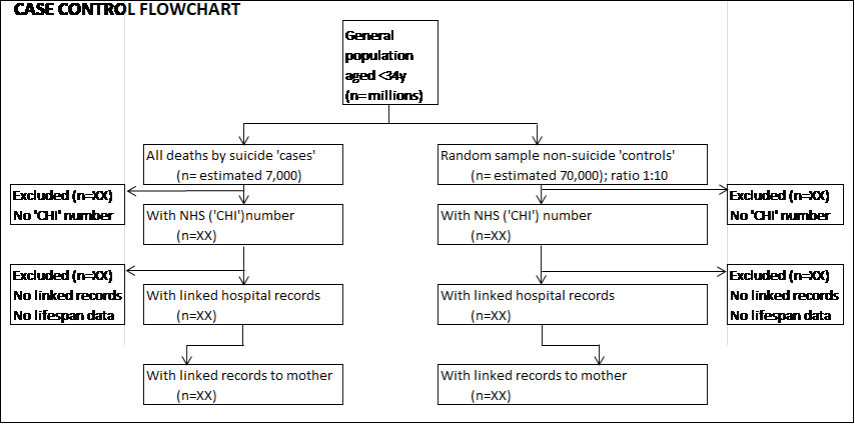


Controls will be selected in a ratio of 1:10 (about 70,000 controls), and alive at the time of death of the cases, therefore the denominator is not a strict measure of disease frequency; however, any odds ratios will be similar to estimates obtained had the whole population been sampled, known as the ‘rare disease assumption’ [21]. A 1:10 ratio is selected as ICD codes for childhood abuse or neglect are highly likely to be underreported. A flowchart will be populated, with reasons for excluded data (Figure 1).

### Operationalising variables for childhood adversity and mental health

Hospital episodes in the Scottish Morbidity Records (SMR) are coded on a main diagnosis and up to ten secondary diagnoses using the World Health Organisation (WHO) version of ICD-9 from 1981 to 1996, with a change to ICD-10 from 1 April 1996 onwards.

Our working definition of childhood adversity draws on four key studies which have reported ICD codes for maltreatment, violence-related (MVR), diagnoses suggestive of maltreatment, and mental-health related diagnoses [17–20]. To define MVR events, ICD-10 codes will be used from González-Izquierdo et al which were derived from National Institutes for Health and Care Excellence (NICE) guidance and expert consultation, and reflecting a hierarchy of maltreatment likelihood [17,18], grouped by Maltreatment, Assault, Undetermined Intent and Adverse social circumstances (indicating neglect or broader welfare concerns, such as homelessness, inadequate housing, etc.) Gilbert et al cross-mapped these same codes to ICD-9 and we will make use of these tabulated cross-mapped codes [18].

We will supplement these ICD codes with a further set of published ICD codes suggestive of child maltreatment, derived from expert consultations and case note review [19]. To complement the underreporting of maltreatment [20], Schnitzer et al conducted a case note review of 2,826 hospital visits of children suspected of maltreatment and found that 1,200 (43%) were confirmed positive for maltreatment, with 68 ICD codes able to classify >66% of visits as maltreatment-related [19]. Codes suggestive of maltreatment included specific fractures, burns, and injuries of undetermined intent, amongst others, as well as upper age limits and exclusion criteria (e.g. a diagnosis of malnourishment without an underlying illness) [19]. These suggestive codes have been previously defined and published in ICD-9-CM (Clinical Modification, an ICD variant developed in the USA). As part of this study, these ICD-9-CM and ICD-10-CM codes will be cross-mapped to the equivalent codes in the unmodified WHO versions of ICD-9 and ICD-10.

Both these MVR codes and codes suggestive of maltreatment were used to estimate population rates, and either used a hierarchy of codes, or reported population weights, respectively. In this study, we are primarily concerned with finding individual-level evidence of maltreatment with maximum sensitivity, so we will first use these studies’ reported inclusion and exclusion codes, without the hierarchy or population weights. This will be followed by an attempt at an estimate of absolute risk as well as relative risk, using this study’s case-control ratio of 1:10 where all relevant ‘cases’ are included (and no inferences made), and using control group characteristics and NRS data to give an inferred estimate of general population equivalent numbers.

To achieve consistent coding of mental health-related conditions over the period of the study, we will use the Clinical Classification Software (CCS) published by the US Agency for Healthcare Research and Quality (AHRQ) [23,24]. CCS is a unique mapping from all ICD-9-CM and ICD-10-CM codes to aggregated categories of injuries and illness, including mental health. As part of this study, these ICD-9-CM codes will be cross-mapped to the equivalent codes in the unmodified WHO versions of ICD-9 and ICD-10.Therefore, in order to define a typology of childhood adversity and mental health, episodes will be flagged as ‘Maltreatment or violence-related’ (MVR), ‘Suggestive of maltreatment’, and/or ‘Mental health-related', depending on the diagnosis codes matching the relevant inclusion and exclusion criteria, and drawing on all the published work above [17–20]. This approach will maximise the sensitivity of the available validated codes, a decision taken in consultation with the SSG, and with consideration to the likely underreporting of childhood adversity in hospital records.

### Additional variables

These datasets are, in turn, to be linked to derived area-level variables based on postcode and held by NHS ISD. For deprivation, the Carstairs Morris Index will be used, the only variable available with data spanning all years from 1981 onwards, based on refreshed Census decade data at postcode level for car ownership, occupational social class, household overcrowding and male unemployment. The Scottish Index of Multiple Deprivation, used from 2000 onwards and the Scottish Government’s official tool for identifying areas with concentrations of deprivation has been requested; however this will likely have limited value having a shorter timespan than the study data. An 8-factor urban-rural indicator derived from postcode and available since 2004 has also been requested; this may also be of limited value as it covers a limited period. A crude geographic variable of NHS Health Board has been requested to explore differences by geographical areas where treatment is accessed.

### Data analysis

Prior to data analysis, extensive cleaning and checking data validity will be done, in line with methods used in previously published work [22]. Data will be assessed for ‘missingness’ before applying descriptive statistics to estimate measures of central tendency and variability, or frequencies and their relative percentages for groups on age, gender, and deprivation quantile; numbers of people exposed to early adverse events, and the type, number and timing of adverse events [RQ1]; numbers who then subsequently develop mental ill-health and/or suicidal behaviour [RQ2]. The number of adverse events and any dose-response relationship with suicidal behaviour will be explored [RQ3]. Maternal records will be summarised for death and maternal ill health in the perinatal period, permitting an analysis of added impact of maternal adversity, should the records be sufficiently populated [RQ4]. Where there are sufficient proportions of linked physical and mental health maternal records available, it may also be possible to explore any inter-generational trauma [RQ4].

Relative risks of certain combinations of predictor variables as per the research questions on the outcome of suicide will be computed. These data may be of sufficient quality to permit odds ratios to be estimated from the use of logistic regression with the dependent variable of death by suicide (‘cases’) or being alive (‘controls’) as an outcome, given certain events have happened to individuals. These events include independent variables such as number, type, and first occurrence of hospital admissions for childhood adverse events (violence and maltreatment), hospitalisations for adversity or mental health, and maternal adverse events including bereavement of mother in childhood. In order to minimise confounding, preventive measures are in place by using controls randomly derived from the general population who are age, gender and geographically matched to those who died by suicide (see Study population). As is the case with observational studies, imbalances in other prognostic factors may be present requiring adjusted analyses. For example, an assessment of the distribution of ICD chapters for physical health diagnoses between groups will be made, in order to identify whether presentation of ICD-10 codes for childhood adversity or mental ill-health are not confounded by an association with physical ill-health. 

### Developing stakeholder-informed potential models of intervention

Three key sources will be used in developing types of interventions for further development, 1) findings from aim 1; 2) existing literature on effectiveness and barriers to implementation in the area and conceptual models of suicide such as the pre-motivational phase of the Integrated Motivational-Volitional Model [25]; and 3) expert opinion from healthcare practitioners, people with lived experience accessing services, other public services, and policy makers. Such an approach is consistent with the recent *Lancet Psychiatry* Commission’s recommendations on the development and implementation of psychological treatments [26]. Workshops guiding the discussion, including dissemination of study findings, will be summarised with areas for future development and research investigation [RQ5]. Workshops will integrate existing evidence, establish feasible models of potential interventions, and conduct final modelling testing via clinical scenarios and with individuals who have real world knowledge.

## Results

### Maltreatment and Violence Related diagnosis codes

The ICD-10 codes from González-Izquierdo et al [17] and ICD-9 codes from Gilbert et al [18], were cross-mapped and tabulated (Table 2). In addition, ICD-9 code E904 ‘Hunger, thirst, exposure, neglect [20], and ICD-10 codes Y87.1 ‘Sequelae of assault’ and Y87.2 ‘Sequelae of events of undetermined intent’ will be included as MVR codes under the Maltreatment, Assault, and Undetermined cause headings, respectively.

**Table 2: Maltreatment or violence-related (MVR) codes compiled from Gilbert et al. (2012) and González-Izquierdo (personal communication, 2019):** 

MVR Category	ICD-9	ICD-10
Maltreatment	994.2 - 994.3, 995.5, E967	T73, T74, Y06, Y07
Assault	E961 - E966, E968 - E969	X85 - Y03, Y04, Y05, Y08 - Y09
Undetermined cause	E980 - E989, V68.2, V70.4, V71.4, V71.5, V71.6, V71.81	Y10 - Y34, Z04.0, Z04.5, Z04.8
Adverse social circumstances	779.5, V15.4, V15.5, V15.89, V15.9, V17.0, V20.0, V20.1, V60, V61, V62.4, V62.5, V62.81, V62.89, V62.9, V69	P96.1, Z58.8, Z58.9, Z59.0, Z59.1, Z59.4, Z59.5, Z59.7, Z59.9, Z60 - Z63, Z64.4, Z65.3, Z65.8, Z65.9, Z72, Z74, Z76.1, Z76.2, Z81, Z86.5, Z91.6, Z91.8

### Cross-mapping between the WHO and the CM variants of ICD

An attempt at automatic, bottom-up mapping between the WHO and CM variants of ICD, finding a unique match between every CM code to a WHO code, proved not to be feasible. In general, the CM variant provides more detailed classifications, with codes up to seven characters long, and more codes in the catalogue in total. Although most such codes can be truncated to the number of characters to match codes in the WHO catalogue, there were several instances where conditions were assigned different 3-character or 4-character codes in the different ICD variants (see Table 3 for an ICD-10 example). In these circumstances, we conducted a systematic manual review of relevant codes and corrected them accordingly.

**Table 3: Example of discrepancy between ICD-10-CM and ICD-10 at 3-character level.** Footnote: * The ICD-10-CM system does not require additional external cause codes for self-poisonings. ICD-10-CM T36-T65 are combination codes that include the substance taken as well as the intent, where *self-harm or **undetermined intent is indicated by 5th or 6th digit code.

	ICD-10-CM	ICD-10
Intentional self-harm	X71-X83; T36-T65*, T71	X60-X84
Assault	X92-Y08	X85-Y09
Event of undetermined intent	Y21-Y33; T36-T65**; T71	Y10-Y34

### Cross mapping between ICD versions 9 and 10

The WHO catalogues for ICD-9 and ICD-10 were downloaded from the UK Biobank (ICD-9: https://biobank.ctsu.ox.ac.uk/crystal/coding.cgi?id=87; ICD10: https://biobank.ctsu.ox.ac.uk/crystal/coding.cgi?id=19), and the online ICD-10 reference (version 2016) provided by the WHO was used for keyword searches (27). The CM variants of ICD-9 and ICD-10 were included in the statistical software ‘R’ package “icd”, version 4.0.6 and available for use (28). The General Equivalence Mapping (version 2018) from ICD-9-CM to ICD-10-CM and vice versa was also used for reference (available at ftp://ftp.cdc.gov/pub/Health_Statistics/NCHS/Publications/ICD10CM/2018/).

### Codes suggestive of maltreatment

For cross-mapping codes suggestive of maltreatment, a piecemeal approach was used: to map from ICD-9-CM to ICD-9, exact code matches were found first and manually reviewed to check for discrepancies. Codes which could not be exactly matched were keyword searched in the ICD-9 catalogue. Three codes could not be matched from ICD-9-CM to a corresponding ICD-9 code and these were: ICD-9-CM Codes E869.4 Second-hand tobacco smoke, V71.81 Observation for abuse/neglect, and 362.81 Retinal haemorrhage.

Next, ICD-9 codes were matched to ICD-10 codes, starting from top-level (3-character) codes where appropriate and narrowing down to the required level of detail, using the code catalogues described above. We have made available the results of cross-mapping as supplementary tables (see Appendix Tables 1-3). Further details of cross-mapping are available from the authors.

### Clinical Classifications Software (CCS)

Mental health categories are numbered 650-670, which also includes suicide and self-harm as a single category, numbered 662. For our analyses, we created a category for ‘Events of undetermined intent’, which spanned several CCS categories. A cross-mapping was performed between the ICD-10-CM codes in CCS to the ICD-10 codes in use in the UK, and between the ICD-9-CM codes and ICD-9. The following versions were used, 1) ICD-9-CM CCS version 2015 [23], available at https://www.hcup-us.ahrq.gov/toolssoftware/ccs/ccs.jsp, and 2) ICD-10-CM CCS beta version 2019.1 [24], available at https://www.hcup-us.ahrq.gov/toolssoftware/ccsr/ccs_refined.jsp.

For ICD-CM codes (versions 9 and 10 separately), exactly matching ICD codes at up to 4-characters deep were found; the remaining unmatched codes were assigned a CCS category if the entire 3-character code mapped to a single category (i.e. all ICD-9 codes starting with 290 are in the dementia/delirium CCS category, whereas 300 Neurotic disorders map onto two CCS categories, Anxiety, and Mood disorders, respectively); the remaining codes were manually mapped to appropriate codes. A full list of codes and details of cross-mapping are available from the authors.

## Discussion

### Main findings

This protocol describes and reports on methods and results of harmonising ICD codes through four decades and between datasets, a labour-intensive process. In an increasingly data-oriented world, data science has potential to be transformative, a means of exploiting rapidly expanding ‘big data’, leading to solutions for known problems [15,29]. Linking longitudinal routine health and research data provide new opportunities for evaluating intervention effectiveness, and establishing unintended consequences [29]. However, this is only feasible provided it is possible to track data through time with consistent codes, that the data are reasonably complete and of sufficient quality. This protocol focuses on the first of these, tracking harmonised ICD codes through time.

Mapping ICD codes between catalogues 9 and 10 was labour-intensive and challenging, requiring expertise in several domains, including data science, statistical programming, longitudinal study design, multiple coding systems and clinical classifications software. Nevertheless, the cross-mapping tasks were less onerous than they otherwise would have been. We were able to draw on experience from previous research [22], relevant statistical programming and cross-mapped coding frameworks published in table format [19], and other frameworks shared via direct communication [17,18]. We add, in turn, to this body of knowledge in providing detailed legacy cross-mapping frameworks, enabling emerging technological approaches to interrogate and predict individuals most at risk from similar longitudinal data. Without openly available information, progress on the preparatory work prior to any analysis would have been much longer and more tedious. 

### Potential benefits and importance of the study

It has been reported that current care pathways for people who present to hospital in distress or with mental health symptoms are sub-optimal. Around 30% of people who have taken their own lives in Scotland have attended Emergency Departments in the three months prior to death [13], and more than half (58%) of deaths which occurred within three months of hospital discharge did so after last discharge from general hospitals, not psychiatric hospitals [22]. The Scottish Ambulance Service transported half of individuals seen as ‘psychiatric emergencies’ in 2011 to general hospital, with an estimated 8% (59/6,802) who were alive after one day of admission taking their own lives in the following year [30]. There is a strong need to overhaul ways of working with an emphasis on whole system multi-agency partnerships between our first responders (paramedics, policing, Emergency Departments), and if interventions are to be effective these need to engage wider sectors of education, local authorities for social care, and charitable organisations.

Suicide is a tragic catastrophic outcome for some people who experience distress and entrapment, as exemplified by the integrated motivational-volitional (IMV) model [25]. Uncovering patterns of hospital contact prior to suicide will provide important contextual information for the IMV model, and it will help to map out opportunities for intervention and provide targets for treatment or support. 

### Strengths and limitations

The main study strength is the longitudinal case-control study design that will provide an opportunity to explore causal mechanisms as identified by hospital contacts along the lifespan for young people who die by suicide. This study will establish whether documented hospital contacts for people who suffer childhood adversity are higher in those with an outcome of suicide compared with the randomly selected proportion of the general population. The study uses de-identified linked data in a secure environment, the national ‘safe haven’ and the data controller remains the NHS, therefore anonymity is preserved.

There are limitations, and these include identifying relatively rare events in routine data, with instances of abuse or maltreatment likely to be under-recorded and underrepresented, or with people not brought to hospital, even if severe. However, this is also likely to be the case in the control dataset and the relative differences between groups will be important. Other limitations are that there is no corresponding paternal data to summarise alongside maternal data, and that as the data span four decades these will have variable data quality and consistency of coding through time. This is particularly relevant to the change in ICD-9 to ICD-10 catalogues in 1996, catalogues not intended to be compatible. Sophisticated methods for cross-mapping ICD catalogues are nevertheless possible, and we will use Clinical Classifications Software algorithms, a technique used successfully in a previous study [22].

Another strength of this study is the planned stakeholder engagement to disseminate and solicit the views of health and public service practitioners, voluntary sector and people with lived experience. Although these multi-agency national workshops will identify opportunities to develop interventions in supporting people earlier in life at increased risk of suicidal behaviour, intervention development will be confined to the early stages only. It is hoped that sufficient detailed information will be available to develop and test a model in a future research study. 

## Conclusion

There is a dearth of evidence about touchpoints prior to suicide. This study reports methods of harmonising ICD codes and their published cross-mappings along the lifespan to understand hospital contact patterns for childhood adversity that come to the attention of healthcare practitioners. Although time-consuming, these harmonised codes are a necessary precursor to the data cleaning and analysis phases of this study and are available for use to other researchers. It is hoped that once analysed, this study will contribute valuable information for the first time on childhood adversity, mental health, and suicidal behaviour related hospital contacts along the lifespan, thus identifying groups to focus earlier identification and intervention, and opportunities for data linkage with other relevant agencies to reveal more insights. 

## Authors’ contributions

ND is the Principal Investigator. ND led the development of the research questions and study design; MM, TK, RO’C, BW, AJ, HC, CF, JB, CH, SA-S, and LN refined the development of the research questions, study design and obtained the funding. ND led obtaining ethical and regulatory approval from the Public Benefit and Privacy Panel (PBPP). ND & JS undertook the cross-mapping exercises. JS is the Research Fellow and will provide analysis and support with project management of the study. GG provided support with literature reviewing. ND will have oversight of the analysis and interpretation of data. All authors provided critical review and final approval of the manuscript, and are in agreement to be accountable for all aspects of the work as determined by disciplinary expertise.

## Consent for publication

Professor Ruth Gilbert, Dr González-Izquierdo et al kindly provided codes extracted in their previous research, and consent has been obtained from González-Izquierdo et al to publish these ICD codes pertaining to Violence and Maltreatment.

## Ethics statement

The study was reviewed and approved by the North of Scotland Research Ethics Committee 1 (REC) on 04 May 2018 with REC reference 18/NS/0054. Consent to use the de-identified data was provided by the Public Benefit and Privacy Panel (PBPP) for Health and Social Care on 6 September 2018 with reference 1617-0228. 

## Supplementary files

Appendix 1 (Supplementary tables 1-3)

## Abbreviations

CHI	Community Health Index
eDRIS	electronic Data Research and Innovation Service
ICD	International Classification of Disease
ISD	Information Services Division
NHS	National Health Service
PBPP	Public Benefit and Privacy Panel
SMR	Scottish Morbidity Record
