# Supplementary material for: Childhood adversity, mental health and suicide (CHASE): a methods protocol for a longitudinal case-control linked data study
Source: Int J Popul Data Sci. 2019 Apr 2;5(1):1338. doi: 10.23889/ijpds.v5i1.1338 (PMC7473285; doi:10.23889/ijpds.v5i1.1338)
Supplement: Appendix 1 (Supplementary tables 1-3) [file ijpds-05-1338-s001.pdf]

Appendix

Supplementary table 1. Cross-mapping of codes suggestive of maltreatment to ICD-9 and ICD-10*

Conditions in ICD-9-CM	Code		Co-occurring exclusion codes	
	ICD-9	ICD-10	ICD-9	ICD-10
Genital herpes	054.1	A60	771.22	P35.2
Gonococcal infection	098	A54	098.4, 771.6	A54.3, P39.1
Pelvic inflammatory disease, unspecified	614.9	N73.9		
Contusion of genital organs	922.4	S30.2	286–287, E800–E819	D65-D69, V01-V99
Observation after alleged rape	V71.5	Z04.4		
Observation for abuse/neglect	N/A	N/A		
Retinal haemorrhage	N/A	H35.6	286–287, E810–E813, E815–E819[1]	D65-D69, V20-V99
Rib fracture	807.0, 807.1	S22.30, S22.31	767, 765, 756.50, 733.1, E810–E813, E815–E819[1]	P10-P15,P52.4,P52.6,P52.8,P52.9, P05,P07, Q78.0, M48.5,M80,M84.4,M90.7, V20-V99
Scapula fracture	811	S42.1	767, 765, 756.50, 733.1, E810–E813, E815–E819[1]	P10-P15,P52.4,P52.6,P52.8,P52.9, P05,P07, Q78.0, M48.5,M80,M84.4,M90.7, V20-V99
Traumatic subdural haemorrhage	852	S06.5	286–287, E810–E813, E815–E819[1]	D65-D69, V20-V99
Other/unspecified intracranial haemorrhage	853.0	S06.8	286–287, E810–E813, E815–E819[1]	D65-D69, V20-V99
Stomach injury	863.1	S36.31	E810–E813, E815–E819[1]	V20-V99
Assault	E965, E966, E968.2	X93-X96, X99, Y00	E960.1, E968.4	Y05,T74.2, Y06
Assault, NOS	E968.9	Y09	E960.1, E968.4	Y05,T74.2, Y06
Undetermined intent, other means	E988	Y19,Y26,Y27, Y31-Y34		
Skull vault fracture	800	S02.0	767, 765, 756.50, 733.1, E810–E813, E815–E819[1]	P10-P15,P52.4,P52.6,P52.8,P52.9, P05,P07, Q78.0, M48.5,M80,M84.4,M90.7, V20-V99
Vertebral fracture	805	S12.0,S12.1, S12.2,S12.7, S12.9,S22.0, S22.1,S32.0, S32.7	767, 765, 756.50, 733.1, E810–E813, E815–E819[1]	P10-P15,P52.4,P52.6,P52.8,P52.9, P05,P07, Q78.0, M48.5,M80,M84.4,M90.7, V20-V99
Traumatic subarachnoid haemorrhage	852	S06.6	286–287, E810–E813, E815–E819[1]	D65-D69, V20-V99

Intrathoracic injury, NEC	862	S277-S279	E810–E813, E815–E819[1]	V20-V99
Small intestine injury	863.2, 863.3	S36.40, S36.41	E810–E813, E815–E819[1]	V20-V99
Spleen injury	865	S36.0	E810–E813, E815–E819[1]	V20-V99
Spinal cord injury	952	S14.0,S14.1, S24.0,S24.1, S34.0,S34.1, T06.0,T06.1, T09.3	E800–E819	V01-V99
Other severe malnutrition	262[2]	E43[2]		
Dental caries	521.0	K02		
Solar radiation dermatitis	692.7	L57.8		
Pelvic fracture	808	S32.1-S32.8,T02.1	767, 765, 756.50, 733.1, E810–E813, E815–E819[1]	P10-P15,P52.4,P52.6,P52.8,P52.9, P05,P07, Q78.0, M48.5,M80,M84.4,M90.7, V20-V99
Traumatic pneumohemothorax	860	S27.0-S27.2	E810–E813, E815–E819[1]	V20-V99
Heart or lung injury	861	S26, S27.3-S27.6	E810–E813, E815–E819[1]	V20-V99
GI injury, NEC	863.8	S36.2,S36.8, S36.9	E810–E813, E815–E819[1]	V20-V99
Liver injury	864	S36.1	E810–E813, E815–E819[1]	V20-V99
Kidney injury	866	S37.0	E810–E813, E815–E819[1]	V20-V99
Burn of head	941	T20	E890–E897	X00-X09
Burn of trunk	942	T21	E890–E897	X00-X09
Burn of leg	945	T24,T25	E890–E897	X00-X09
Burn of multiple sites	946	T29	E890–E897	X00-X09
Poisoning by drugs/medicinals	960-979	T36-T50	E870–E876	Y60-Y69
Drowning, non-fatal submersion	994.1	T751		
Second-hand tobacco smoke	N/A	Z58.7[3]		
Swimming accident	E910.2	W67-W70		
Bathtub (near) drowning	E910.4	W65,W66		
Other (near) drowning	E910.8	W73		
Accidental (near) drowning, NOS	E910.9	W74		
Unarmed fight, brawl	E960.0	Y04	E960.1, E968.4	Y05,T74.2, Y06
Undetermined intent, poisoning	E980	Y10-Y19		
Undetermined intent, firearm	E985	Y22-Y25		
Household circumstances	V60	Z59		

Footnotes: *Codes extracted from Schnitzer PG, Slusher PL, Kruse RL, Tarleton MM. Identification of ICD codes suggestive of child maltreatment. Child Abuse & Neglect. 2011 Jan 1;35(1):3–17; [1] Excluding codes where 4th digit is .6 or .7; [2] For exclusion codes see Supplementary Table 2; [3] Include only if any codes in Supplementary Table 3 are also present.

Supplementary Table 2. Exclusion codes for use with ‘severe’ malnutrition codes.

ICD-9	ICD-10
009.0	A09
010-018	A15-A19
042-044	B20-B24
070	B15-B19
140-208	C00-C97
243-244	E00-E03,E89.0
250	E10-E14
252	E20-E21, E89.2
253	E22-E23, E89.3
270-275	E70-E90, D89, M10
271.3	E73
277.0	E84
317–319	F70-F79
330-344	G00-G99, R52
431	I61
446	M30-M31
493	J45
530.8	K21
555-558	K50-K52
575-576	K82-K83
571	K70-K76
577.8	K86.8
579	K90-K91
588.8	N25.8
593.9, 585.9,	N18-N19, N28.9
599.0	N39.0
710	M32-M36
714	M05-M06, M08, M12.0
745-747	Q20-Q28, P29.3
749	Q35-Q37
750.5	Q40.0
751.3	Q43.1
758	Q90-Q99
760.76	Q86.0, P04.3
767.0	P10, P11.1, P11.2
770.7	P27.1
771	P35-P39
772.1	P52
852-853	S063-S068
984	T56.0

Supplementary Table 3: Additional inclusions required for use with second-hand smoke code.

ICD-9	ICD-10
480-487	J10-J18
490,491	J40-J42
466	J20-J21,J68
493	J44-J46
381.0–381.4	H65
